# Snakebite and Its Socio-Economic Impact on the Rural Population of Tamil Nadu, India

**DOI:** 10.1371/journal.pone.0080090

**Published:** 2013-11-21

**Authors:** Sakthivel Vaiyapuri, Rajendran Vaiyapuri, Rajesh Ashokan, Karthikeyan Ramasamy, Kameshwaran Nattamaisundar, Anburaj Jeyaraj, Viswanathan Chandran, Prabu Gajjeraman, M. Fazil Baksh, Jonathan M. Gibbins, E. Gail Hutchinson

**Affiliations:** 1 Institute for Cardiovascular and Metabolic Research, School of Biological Sciences, University of Reading, Reading, United Kingdom; 2 School of Chemistry, Food and Pharmacy, University of Reading, Reading, United Kingdom; 3 Department of Biotechnology, Karpagam University, Coimbatore, Tamil Nadu, India; 4 Department of Mathematics and Statistics, School of Mathematical and Physical Sciences, University of Reading, Reading, United Kingdom; Tulane University School of Public Health and Tropical Medicine, United States of America

## Abstract

**Background:**

Snakebite represents a significant health issue worldwide, affecting several million people each year with as many as 95,000 deaths. India is considered to be the country most affected, but much remains unknown about snakebite incidence in this country, its socio-economic impact and how snakebite management could be improved.

**Methods/Principal Findings:**

We conducted a study within rural villages in Tamil Nadu, India, which combines a household survey (28,494 people) of snakebite incidence with a more detailed survey of victims in order to understand the health and socio-economic effects of the bite, the treatments obtained and their views about future improvements. Our survey suggests that snakebite incidence is higher than previously reported. 3.9% of those surveyed had suffered from snakebite and the number of deaths corresponds to 0.45% of the population. The socio-economic impact of this is very considerable in terms of the treatment costs and the long-term effects on the health and ability of survivors to work. To reduce this, the victims recommended improvements to the accessibility and affordability of antivenom treatment.

**Conclusions:**

Snakebite has a considerable and disproportionate impact on rural populations, particularly in South Asia. This study provides an incentive for researchers and the public to work together to reduce the incidence and improve the outcomes for snake bite victims and their families.

## Introduction

Snakebites represent a significant health issue worldwide, estimated to affect several million people each year [1], [2], and has been estimated to result in 95,000–150,000 deaths [1] annually. Despite this it has only recently been officially recognised as a neglected tropical disease by the World Health Organization. The problems associated with snakebite are particularly acute in South Asia, and India in particular, which is considered to have the highest incidence of snakebites and associated deaths in the world [2]–[4].

Much remains unknown about snakebites in India. Knowledge about the snakes responsible is still developing: the major snakes of medical importance in India have historically been considered to be: the Russell’s viper (*Daboia russelii*), the saw-scaled viper (*Echis carinatus*), the Indian cobra (*Naja naja*) and the common krait (*Bungarus caeruleus*), which together are known as the ‘Big Four’. However, other snakes such as the hump nosed pit-viper (*Hypnale hypnale*), the Levantine viper (*Macrovipera lebetina*) and others [5]–[8] are now also considered to be medically relevant. The Indian government has recently adopted the World Health Organization’s model [9] for defining snakes of medical significance, which will mean that the range of snakes recognised to be responsible for injury and death in India will continue to grow. These issues have consequences for snakebite management: the current treatment in rural India remains polyvalent antivenom raised against venom from the Big Four snakes only. The effectiveness of this against bites from snakes not in the Big Four group, and even against snakes from different geographical regions is unclear. Furthermore, use of antivenom in cases where it is not effective or not needed (e.g. bite from a non venomous snake) is both expensive and potentially dangerous to the victim because of the possibility of anaphylactic reactions. Thus there is a need to improve diagnosis of snakebite and to develop new treatments which have reduced side effects and are effective against snakes other than the Big Four too. Information about snakebite incidence is also lacking: there is insufficient epidemiological data, particularly in the rural areas where snakebites are most common. Snakebite morbidity and mortality are generally considered to be under-reported, largely because not all victims are treated in hospitals [3], [10]–[13]. Community surveys are considered to be a vital means for obtaining reliable estimates of the true incidence and impact of snakebites [4], [12], [14], [15].

In this study our objective was to obtain a more complete understanding of the incidence and effects of snakebites among the rural population of India. In particular, this study was aimed to obtain the snakebite incidence rate in three different sizes of rural villages, prevalence rate in male and female populations, and socio-economic impact of snakebites on rural population. Hence, we have conducted a study within the Indian state of Tamil Nadu. This combined a household survey of snakebite incidence in 30 villages (28,494 people) with detailed interviews with victims or their families to obtain information about the circumstances, treatment and socio-economic effects of the snakebite. We believe this is the first time that a snakebite study in India has involved members of a community living with the risk of snakebite and victims who have experienced snakebites. The results highlight the impact of snakebite on rural populations and major issues in its management, and will provide a useful basis for developing improvements to snakebite management in India and other countries in South Asia.

## Methods

### Ethical Statement

This research was conducted according to the Declaration of Helsinki and the ethical guidelines of the Indian Council of Medical Research. The research and the consent forms and questionnaire for victims (Study material S1) were approved by the research ethics committee of the School of Biological Sciences, University of Reading. Surveys were conducted between November and December 2010 in India and permission was obtained from village and Panchayat leaders. The aims of the research were explained to the participants in local languages and informed written consent was obtained from all study participants. All data were anonymised prior to analysis.

### Household Survey

Household surveys were conducted within the Indian state of Tamil Nadu because of the easy accessibility and familiarity to the authors. Villages were divided into three categories based on the number of households, as reported in the 2001 Census of Tamil Nadu [16]. Type I villages had fewer than 100 houses, Type II villages had between 100 and 250 houses and Type III villages had more than 250 houses. This type of village categorisation is based on the accessibility for snakes to enter the villages. For example, due to the small number of households and reduced activity in Type I villages, the snakes may more easily enter the villages and cause increased numbers of bites, compared to the larger villages where more households with increased activity may restrict the freedom of snakes in villages. Moreover, the majority of population in smaller villages are involved in agricultural work. Ten villages from each of the three categories were randomly selected based upon population sizes estimated to yield statistically meaningful data while maintaining a size of study that was practically possible. Further investigation showed that the sampled villages adequately represented the different geographical regions found in Tamil Nadu. The questionnaire was pretested prior to data collection for its appropriate design and all the field investigators were trained to ensure appropriate and uniform approaches during interviews. Household surveys were conducted from every house in each sampled village, a total of 7,578 households representing 28,494 people, to collect information about the population (number of members in the family, their sex and age groups), occupation and snakebite incidence. In most cases, the head of the family was interviewed but in his/her absence another adult member of the family was interviewed to obtain the relevant information on their family. Within the study villages, no refusal (i.e. 100% response) from respondents to give relevant information was received. Every snakebite incidence that occurred in the last 10 years was cross verified by analysing the relevant medical records from the family, traditional healers and hospitals where they obtained the treatments. Further verification was also performed, where possible, from neighbours and relatives in order to determine the year of bite within last 10 years and to avoid recall bias over this period. Cross verification also aided in validating the data collected. The exact month in which the snakebite occurred during 2010 was clearly documented in order to correlate with the rainfall statistics in 2010.

### Survey of Victims

A more detailed questionnaire (Study material S1) was devised to ask victims about the circumstances of the snakebite, the method(s) of treatments obtained and the socio-economic impacts (direct and indirect issues) the bite has caused to the victim and his/her family. This questionnaire was devised in English and translated into Tamil before interviews. The questionnaire was also back translated into English to see if the translation was appropriate. The questionnaire was pretested prior to data collection for its appropriate design and all the field investigators were trained to ensure appropriate and uniform approaches during interviews. All the information was collected by face to face interviews and no refusal (i.e. 100% response) from the respondents to attend the interviews was received. A breakdown of the direct costs involved during the snakebite treatment and the economic loss for the family was also obtained. Answers were collected from 93 victims and 12 relatives of victims who had died following the snakebite. Between these 105 people this accounted for 129 bites. The interviewees were identified blindly from the 30 sampled villages where the household survey was performed. The interviews were recorded in the local language and later transcribed by the authors.

### Statistical Analysis

The villages in Tamil Nadu were classified into three strata and ten sampling villages were chosen from each stratum. The information collected about the prevalence of snakebites was used to estimate stratum-specific period prevalence rates [within 10 years (2001–2010)] and 95% confidence intervals per 1000 head of population. All estimated stratum-specific characteristics were weighted and adjusted using formulae [17] for simple one-stage cluster samples. The total number of individuals observed within a group was taken as the denominator in the estimation of incidence for that particular group. All the estimates for children under 10 years old were weighted for age. Statistical comparisons of proportions were based on the asymptotic normality of the distributions of estimates and independence between strata; reported values of the test statistic follow a chi square distribution on one degree of freedom. Annual snakebite numbers were reported as cumulative incidents for each year. Annual and monthly incidents were compared with rainfall statistics for the Tamil Nadu state obtained from the Department of Climate and Rainfall, Government of Tamil Nadu [18] and the Hydromet division of the Indian Meterological Department [19]. All the statistical analyses were performed using SPSS statistical package (IBM, USA) and R (http://www.r-project.org/).

## Results

### Household Survey

As described in methods, the villages in Tamil Nadu were classified into three categories (type I, II and III) based on the number of households, and ten villages from each category were randomly chosen for the survey. In total, 7578 households representing 28,494 people were surveyed from these sampling villages. Samples of 621, 1871 and 5086 households were surveyed from Type I, Type II and Type III villages, respectively. The characteristics of the study population are shown in Table 1. The majority of the people surveyed (88.6%) were involved in agriculture. In the type I villages this percentage was slightly higher, 93% and statistically significantly different from the percentage in the moderately sized type II villages (χ^2^ = 11.1, p = 8.3×10^−4^) and larger type III villages (χ^2^ = 21.3, p = 3.8×10^−6^). Although the people who are engaged in agricultural work are at higher risk of snakebites, the remaining population was also considered at risk for snakebite since they live in the villages where snakes can freely enter/survive.

**Table 1:** Characteristics of the sample population.

	*Village type*		
	I	II	III
**Characteristics of the sample population**			
No. of households	621	1,871	5,086
***Sample***			
Male	1,194 (50.6%)	3,515 (50.5%)	9,636 (50.3%)
Female	1,165 (49.4%)	3,451 (49.5%)	9,533 (49.7%)
Total	2,359 (100%)	6,966 (100%)	19,169 (100%)
***Age***			
0–10	319 (13.5%)	992 (14.2%)	2,799 (14.6%)
11–20	469 (19.9%)	1,334 (19.2%)	3,661 (19.1%)
21–30	406 (17.2%)	1,349 (19.4%)	3,583 (18.7%)
31–40	455 (19.3%)	1,268 (18.2%)	3,529 (18.4%)
41–50	377 (16.0%)	1,031 (14.8%)	2,862 (14.9%)
51–60	166 (7.0%)	504 (7.2%)	1,418 (7.4%)
>60	167 (7.1%)	488 (7.0%)	1,317 (6.9%)
***Occupation***			
* Agricultural*	*2,195*	*6,069*	*16,972*
Estimated %	93% (92.1 to 94)	87.1% (83.8 to 90.5)	88.5% (86.9 to 90.2)
* Non-agricultural*	*164*	*897*	*2,197*
Estimated %	7% (6 to 7.9)	12.9% (9.5 to 16.2)	11.5% (9.8 to 13.1)
**Snakebite incidence**			
* No. of snakebites*	*212*	*319*	*878*
Period prevalence per 1000 (95% CI)	90 (77 to 103)	46 (39 to 53)	46 (34 to 57)
***No. of snakebite victims***			
* Male*	*107 (9.0%)*	*159 (4.5%)*	*426 (4.4%)*
Period prevalence per 1000 (95% CI)	90 (76 to 103)	45 (41 to 49)	44 (34 to 54)
* Female*	*60 (5.2%)*	*100 (2.9%)*	*263 (2.8%)*
Period prevalence per 1000 (95% CI)	52 (43 to 60)	29 (19 to 39)	28 (16 to 40)
* Total*	*167 (7.1%)*	*259 (3.7%)*	*689 (3.6%)*
Period prevalence per 1000 (95% CI)	71 (63 to 79)	37 (31 to 44)	36 (25 to 47)
* People with >1 bite*	*31*	*41*	*150*
Period prevalence per 1000 (95% CI)	13 (10 to 17)	6 (4 to 7)	8 (7 to 9)
***No. of snakebite deaths***			
* Male*	*13 (1.1%)*	*24 (0.68%)*	*48 (0.49%)*
Period prevalence per 1000 (95% CI)	11 (8 to 14)	7 (6 to 8)	5 (3 to 7)
* Female*	*5 (0.43%)*	*9 (0.26%)*	*28 (0.29%)*
Period prevalence per 1000 (95% CI)	4 (2 to 7)	3 (1 to 4)	3 (2 to 4)
* Total*	*18 (0.76%)*	*33 (0.47%)*	*76 (0.39%)*
Period prevalence per 1000 (95% CI)	8 (6 to 9)	5 (4 to 6)	4 (2 to 6)

Types I, II and III villages have <100, 100–250 and >250 houses respectively. The percentages in each case were calculated relative to the total population in each type of village. For the snakebite prevalence the percentages indicate the % of the male, female and total population in each village type who suffered snakebites and who died due to snakebite. This data was obtained from the 30 sampling villages.

The total number of snakebites experienced within the villages surveyed was 1409. 1115 people (3.9% of the sample) had been bitten by a snake and 20% of these had been bitten more than once. The rate was higher among men than women: 4.8% of the males sampled had been affected compared with 3.0% of the females. Nine per cent (127 people) of the total bites resulted in death. The number of deaths recorded corresponds to 0.45% of the population of sampled villages. The prevalence (within 10 years) rate per 1000 was calculated as 90 (95% CI 77 to 103), 46 (95% CI 39 to 53) and 46 (95% CI 34 to 57) for type I, II and III villages respectively. People in the small (type I) villages were more likely to suffer from snakebites than in the moderate (χ^2^ = 32.4, p = 1.25×10^−8^) and larger (χ^2^ = 23.8, p = 1.04×10^−6^) villages. Additionally, the number of bites among men (9%) was significantly larger than the number among women (5.2%) in the small villages (χ^2^ = 17.7, p = 9.9×10^−6^). This may link to the higher proportion of agricultural workers in these villages and to the easier access for snakes to small villages compared to larger villages. The death rate in small villages was slightly higher among male (1.1%) than female (0.43%) victims (χ^2^ = 22.2, p = 2.41×10^−6^).

**Figure 1:**
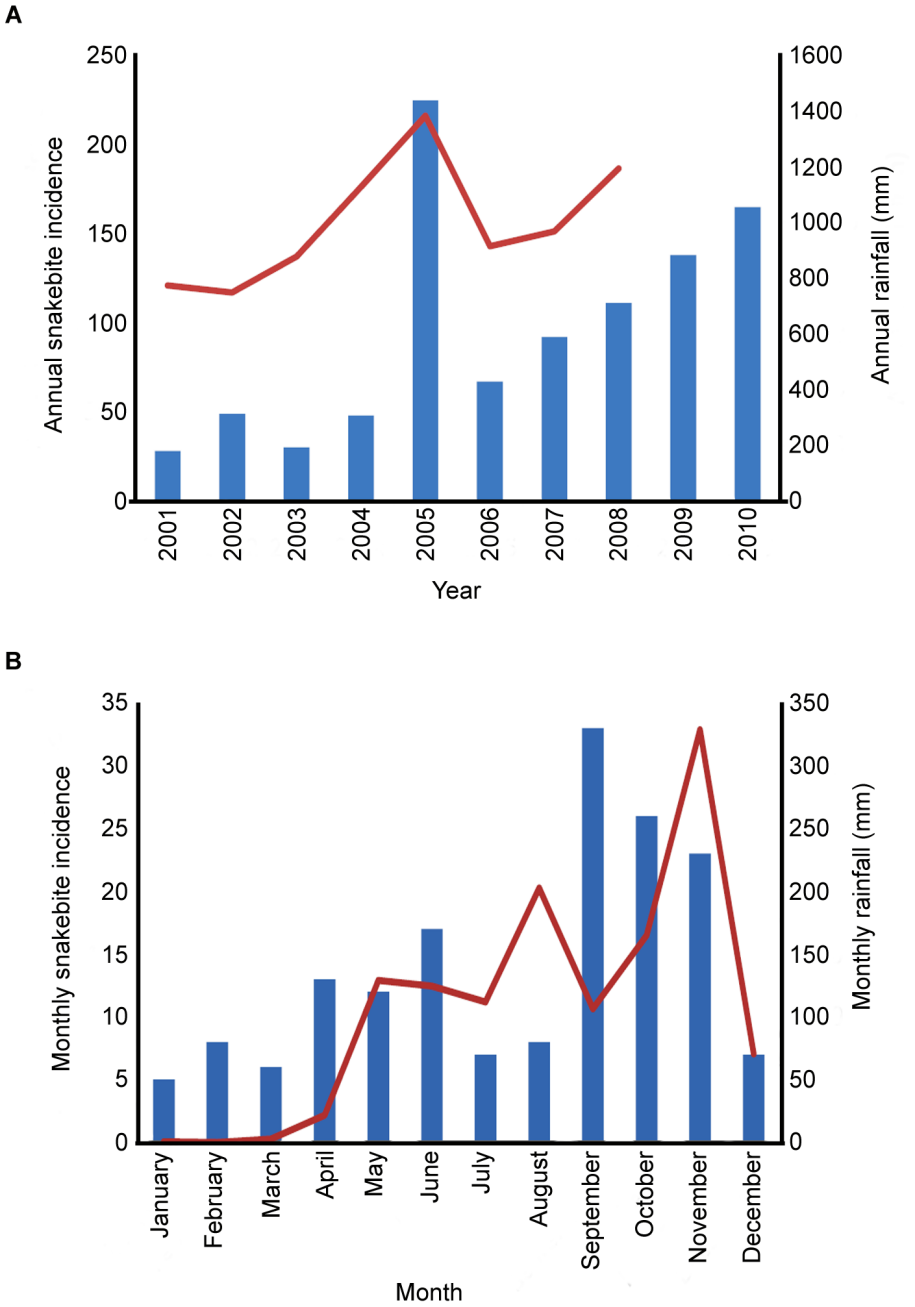
Correlation between rainfall and snakebite incidence. *A*. Annual snakebite incidence (blue bars) and rainfall statistics (red line) for Tamil Nadu from 2001–2010 (obtained from the Department of Climate and Rainfall, Government of Tamil Nadu). The correlation coefficient between the number of bites and rainfall is 0.84, the correlation coefficient between number of deaths and rainfall is 0.87 (data not shown). *B*. Monthly snake bite incidence (blue bars) and average rainfall (red line) for Tamil Nadu in 2010. The monthly rainfall data were obtained from the Hydromet division of the Indian Meteorological Department. The correlation coefficient between the monthly snake bite distribution and the distribution of rainfall is 0.5.

Data regarding the year of snakebite incidences in the last 10 years was checked using more than one mode of cross verification as described in the methods, to avoid inaccurate recollection. On average there were 95 bites per year and 9.1 deaths in the villages surveyed (the number of snake-bite incidents for each year are shown in Table 2). The year-to-year variation correlated with the annual rainfall statistics for the region; more bites and deaths were recorded in years with higher rainfall (Figure 1A). It was possible to collect more precise data (monthly) regarding snakebite incidence during the year in which the study was conducted (2010). The number of snakebites was found to vary within 2010, with the highest number of incidents between September and November, high incidents between April and June and low number of incidents between December and March (Figure 1B). This correlates with the rainfall distribution for Tamil Nadu, and the higher number of incidents also coincides with the months in which increased agricultural activities occur such as crop harvesting (April to June and September to October). During wet months, more snakes may also enter into living areas to capture prey resulting in a greater number of bites.

**Table 2:** Year-wise snakebites and death summary.

	*Village type*				
	*I*	*II*	*III*	*Total*	*Total*
Year	No. of snakebites				No. of deaths
2010	28	37	100	165	13
2009	20	33	85	138	12
2008	14	25	72	111	11
2007	13	21	58	92	10
2006	11	14	42	67	8
2005	30	51	144	225	22
2004	9	11	28	48	6
2003	6	7	17	30	3
2002	9	12	28	49	4
2001	4	8	16	28	2

From the study population, the information about the year of snakebite was obtained from the household members. The information obtained is presented accordingly for each type of study village.

**Figure 2:**
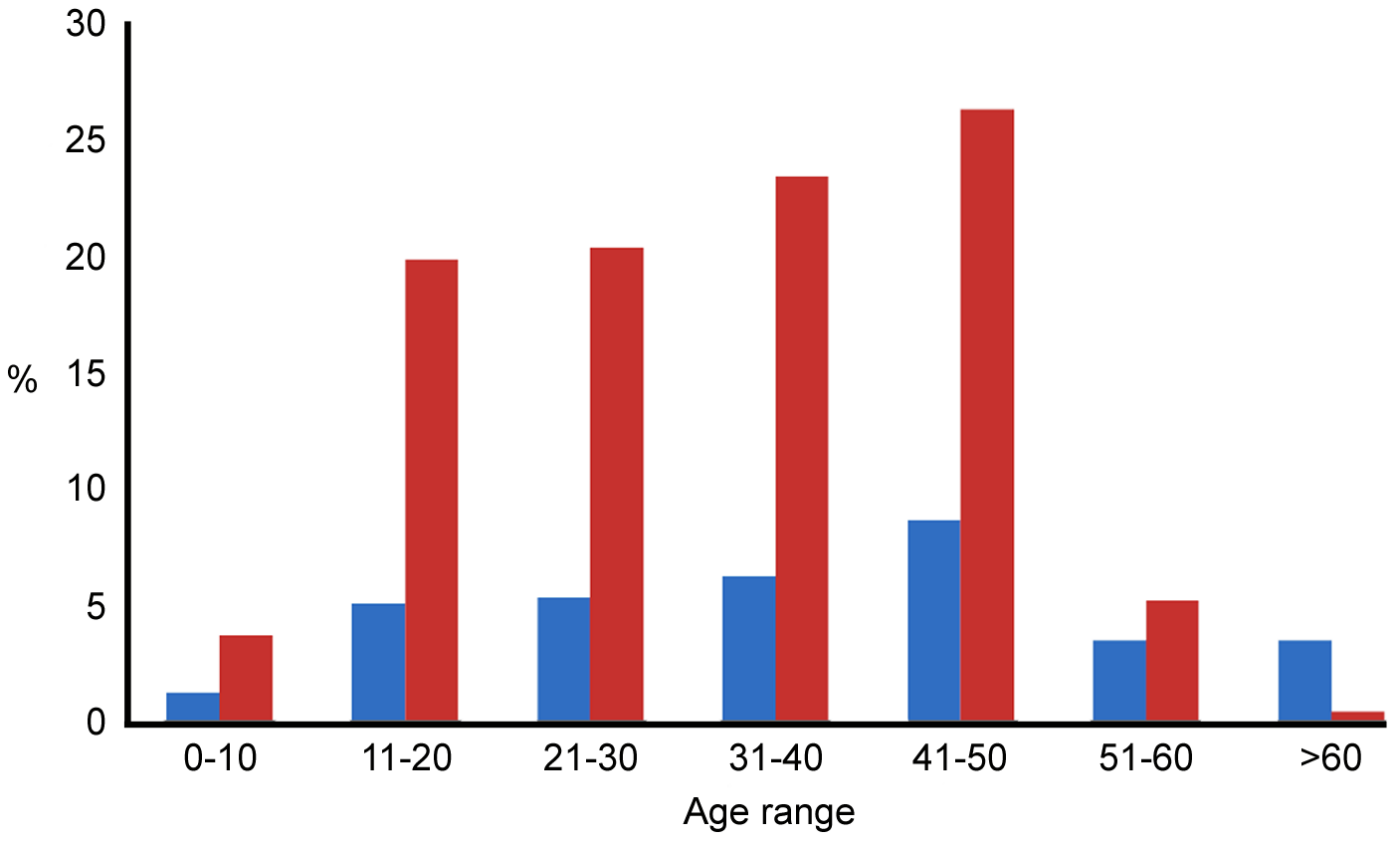
Distribution of snake bites by age group. The red bars show the % of the total number of people which are in each age group identified in the study population. The blue bars show the % of the population of that age group who have been bitten by snakes.

**Figure 3:**
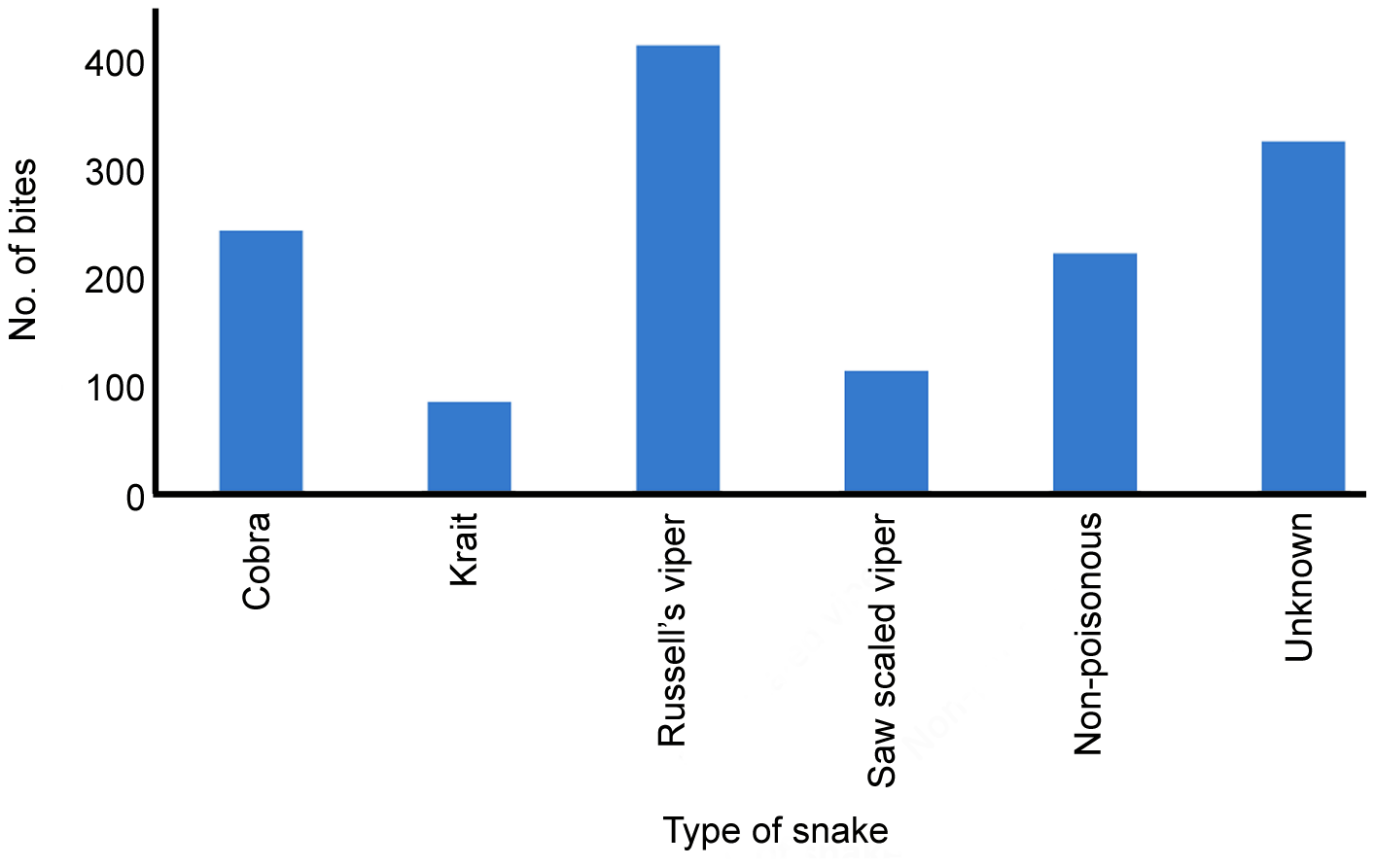
Distribution of snake bites by type of snake. Where the snake species was not identified due to the inability of people to identify the snake, or the bite occurred in dark, these are classified as ‘unknown’.

The graph (Figure 2) reporting the relationship between snakebite prevalence and different age groups shows an increasing snakebite trend in economically active age groups (between 11 and 50). The data are consistent with higher snakebite risk being associated with age groups (40 to 50) more likely to be engaged in agricultural work, while the least affected groups would tend to have a more home-based lifestyle (Figure 2). Only in 77% of cases in the sample was the snake identified by the victim or family. Of these, 79.4% were due to venomous snakes, all of which were the historically recognised Big Four snakes, with Russell’s viper and cobra being the most frequent cause of bites (Figure 3). People living in rural areas are more familiar with, and able to identify the Big Four snakes (cobra, Russell’s viper, saw scaled viper and krait) and some non-poisonous snakes in daylight. But it was not possible to identify the snakes at night time and also some people were not aware of the characteristics of particular snakes (species). Thus, a large proportion of bites (>300) were categorised as of unknown origin (Figure 3).

### Detailed Survey of Snakebite Victims

129 snakebite victims or their relatives answered the more detailed questionnaire which investigated the circumstances of the snakebite incidents, the treatments obtained, and their views about future improvement in treatment provision. In 12 of these cases the victim had died following the bite. Over 79% of the bites occurred when the victims were in the fields and around 15% of bites occurred when they were indoors (Table 3). Over 72% of bites occurred while the victims were working, with a further 19% occurring while the victims were walking along the streets and main roads to villages or agricultural land (Table 3). Consistent with these results, the distribution of bites throughout a typical day shows peaks in the morning (8 am–12 pm) and late afternoon (4–8 pm), times when people would either be at work or travelling to and from work (Table 3). The majority of bites occurred on the lower limbs; over 82% occurred on parts of the leg and 16% on parts of the arm (Table 3) consistent with the most accessible parts of the body. In 20% of the cases, the snakes were killed and 14% of victims took the snake to hospitals for identification. Although, in some cases (16%) the victims did not suffer after the snakebite, in 84% of cases trauma such as severe pain at the bite site, bleeding, giddiness, vomiting, sweating or unconsciousness was experienced. In addition, a small number (7%) of victims were paralysed.

**Table 3:** Circumstances of snakebites and their socio-economic impacts.

*Place of bite*	Frequency	*Person’s activity when bitten*	Frequency
Field	102(79.1%)	Sitting	4 (3.1%)
House	20 (15.5%)	Sleeping	7 (5.4%)
Road	4 (3.1%)	Walking	25 (19.4%)
Outside toilet	3 (2.3%)	Working	93 (72.1%)
***Time of day***		***Part of body bitten***	
00∶00–02∶00	3 (2.3%)	Neck	1 (0.8%)
02∶00–04∶00	0 (0.0%)	Chest	1 (0.8%)
04∶00–06∶00	8 (6.2%)	Forearm	8 (6.2%)
06∶00–08∶00	4 (3.1%)	Hand, fingers	13 (10.1%)
08∶00–10∶00	13 (10.1%)	Hip	2 (1.6%)
10∶00–12∶00	23 (17.8%)	Leg	73 (56.6%)
12∶00–14∶00	6 (4.7%)	Ankle	14 (10.9%)
14∶00–16∶00	9 (7.0%)	Foot	17 (13.2%)
16∶00–18∶00	23 (17.8%)	***Further treatment***	
18∶00–20∶00	18 (14.0%)	None	7 (5.4%)
20∶00–22∶00	11 (8.5%)	Hospital only	86 (66.7%)
22∶00–24∶00	3 (2.3%)	Traditional only	23 (17.8%)
unknown	8 (6.2%)	Traditional and Hospital	13 (10.1%)
***First aid treatment***		Victims received anti-venom	91 (70.5%)
No first aid	83 (64.3%)	***Length of stay for treatments***	
Tourniquet	15 (11.6%)	Less than a week	84 (65.1%)
Tourniquet & incision	12 (9.3%)	1–4 weeks	26 (20.2%)
Incision	1 (0.8%)	1–3 months	19 (14.7%)
Incision and sucking blood	3 (2.3%)	***Length of work leave***	
Calcium carbonate	5 (3.9%)	None	7 (5.4%)
Secretion of *Calotropis gigantea*	7 (5.4%)	Less than a month	53 (41.1%)
Carrying weight	2 (1.6%)	1–6 months	61 (47.3%)
Forced vomiting	1 (0.8%)	More than 6 months	8 (6.2%)
***Treatment cost (rupees/£)***		***Economic loss (rupees/£)***	
0	21 (16.3%)	None	60 (46.5%)
1–1,000 (£0–14)	22 (17.1%)	Jewelry,10000–100000 (£133–1333)	18 (14%)
1,001–5,000/(£14–69)	44 (34.1%)	Crops, 1000–20000 (£14–267)	23 (17.8%)
5,001–10,000/(£69–139)	10 (7.8%)	Cattle, 5000–30000 (£67–400)	12 (9.3%)
10,001–50,000/(£139–664)	18 (14.0%)	Vehicles, 1000–20000 (£14–267)	7 (5.4%)
50,001–350,000/(£694–4858)	11 (8.5%)	Land, 50000–400000 (£667–5333)	5 (3.9%)
Unknown	3 (2.3%)	Lost education	4 (3.1%)

The circumstances of snakebite such as where and when the bite occurred, the activities of victims during bite and the place of bite on the body were obtained from the victims. In addition, the direct costs involved in the treatment of snakebites and their socio-economic impacts were also assessed. The information provided here was from 129 interviewed victims and percentages were calculated accordingly.

In most cases (64.3%) the victims received no first aid immediately after the bite. Where first aid was provided the most common treatment was a tourniquet, applied with or without incision of the wound. 5% of cases were treated with the milky secretion of *Calotropis gigantea*, a traditional medicinal plant which is native to India (Table 3). In certain cases, blood sucking or applying calcium carbonate at the bite site was attempted. In addition, some of the victims were advised to carry heavy weights (believing that it would avoid spreading the venom to the body) and forced to vomit through administration of soap/detergent and/or tamarind solutions orally. In most cases (>98%), initial first aid was given by untrained individuals without any knowledge of snakebites and relevant first aid. In almost 95% of cases the victims sought some form of further treatment: 67% of victims went to hospital, 17% obtained traditional treatments such as extracts from a variety of locally available plants and 10% had both hospital and traditional treatments (Table 3). During hospital treatment, 70% of victims received anti-snake venom (ASV) treatment.

When asked for their views on how treatment following snakebite could be improved, most of the victims or their relatives considered that health care facilities equipped with ASV should be available in each village or, failing that, a vehicle available in each village to take snakebite victims to hospital. Primary health centres were available in some of the villages, but these did not hold any ASV. Despite the cultural emphasis on traditional healers, most victims or their relatives that were questioned would be willing to discontinue this if hospital treatments were easily available. ASV is available free of charge in government hospitals, but the majority of victims suggested that snakebite should be treated free of charge even in private hospitals. Victims also suggested that increased knowledge among the general public about the correct first aid for snakebites and how to handle bites from non-venomous snakes would be a priority.

### Socio-economic Impacts Caused by Snakebites

The snakebite victims and/or their relatives were asked to provide a greater level of detail regarding the socio-economic impacts of the snakebite for them. The major impact caused by snakebite was the financial burden to the family. The direct cost (transport and medical expenses) to the victims of treating the snakebite varied considerably, from as little as zero (16.3% of victims) to a maximum of Rs350,000 (£4,858) (Table 3). The zero cost presumably occurred in cases where either treatment was obtained from Government hospitals or no treatment was obtained. The cost of treatment was increased dramatically in private hospitals due to the severity of the bites and the need for emergency medical equipment such as ventilators. In total, 75% of victims that obtained hospital treatment attended only private hospitals and paid the treatment costs themselves. This cost may also have been increased due to late arrival to multi-speciality hospitals, and consequently increased levels of complications. Delay in hospital treatment may have also been due to having first sought treatments by traditional healers. Similarly, several victims received medical attention at their village primary health centres prior to referral to the nearest town hospitals, and then were further referred to district government hospitals. In some cases they had travelled further to private hospitals.

Since the snakebite is an unexpected incident, the immediate financial pressure depending on the severity of bite, may be substantial. None of the 108 victims who paid for their treatment were covered by medical insurance. Over 40% of victims required to take out a loan to pay for the treatment, and, in order to repay the loan, the families often had to sell their valuables. The financial implications of snakebites were exacerbated by a lack of availability of loans for medical and associated expenses by the nationalised banks. Indeed, 17.8% of victims who paid for their treatment found it necessary to sell stored crops (valued from Rs1000–20000), 14% sold valuable items (valued from Rs10000–100,000), 9.3% sold cattle (valued from Rs5000–Rs30000), 5.4% sold vehicles such as bicycles (valued from Rs1000–2000) and motorcycles (valued from Rs5000–20000), and a small number of people found it necessary to remove their children from education and send them to work, or to sell family land or property (valued from Rs50000–400,000) (Table 3). According to the Indian labour bureau [20] the average daily wage in India for agricultural occupations in 2007–2008 was Rs76 for a man and Rs54 for a woman, thus even Rs1000 represents around half a month’s salary and Rs350,000 (Table 3) represents over 12 years’ salary for a typical agricultural worker. The financial implications may in some cases affect the likelihood of seeking medical attention for snakebites which lead to long term issues associated with morbidity and mortality.

Although the majority (65%) of victims stayed in hospital for less than a week, a considerable number of victims (35%) were admitted for more than a week for their treatment (Table 3), exacerbating the financial hardship (from Rs1000–16000) due to lack of income during this period. In addition, around 50% of victims had home rest after their treatment of between 1 month and 2 years (Table 3), reducing family income (from approximately Rs2000–100,000).

Longer term economic and physical effects are associated with envenomation. In two cases encountered in this study, the bite killed the only son within a family, leaving elderly parents with no financial support. Even where the victims survived there were medium and long term consequences in 90% of cases. In the longer term 68% of victims experienced tiredness, which affected their ability to work as before and thus had to appoint substitutes to work in the fields; in six of these cases the victim was no longer able to work in agriculture and had to find an alternative employment. Many victims (35%) experienced pain, either at the bite site or elsewhere in the body. Other long-term symptoms reported were numbness, swelling of face, hands and legs, liquid oozing from the bite site, blurred vision, eye watering, giddiness, shivering and nausea. In many cases several of these symptoms were reported. These data paint a grim picture of the physical and socio-economic impact of snakebite on the victims and their families.

## Discussion

We have conducted the first large household survey of snakebite incidence in rural Tamil Nadu, India, sampling small, medium and large-sized villages in order to obtain details about snakebites in each type of villages. Our results confirm that snakebite is a significant problem within the rural population, particularly in the smallest villages due to the increased agricultural activities and easy access for snakes. The distribution of bites with respect to age and gender are consistent with snakebite being an occupational health hazard affecting mostly agricultural workers. Bites are more common during periods of high rainfall (most likely due to the migration of snakes into the villages) and at harvest times (due to increased agricultural activity). In most cases the species of snake could be identified and was either one of the Big Four venomous snakes considered to be responsible for most of the bites in South Asia, or a non-venomous snake. However, in a proportion of cases it was not possible for the victim to identify the species. Further snakes have been shown to be of medical significance in this region, but none of these was identified in our survey. This possibly reflects less frequent bites by these snakes or less experience by victims in their identification.

Accepting the limitation in extrapolating these data to the whole population, if we assumed the data obtained from the 30 sampled villages as representative of entire rural Tamil Nadu, we would estimate that around 113,000 snakebites and 10,000 associated deaths occur annually within the rural population of Tamil Nadu. It should be noted that these estimates represent annual averages and that figures will be higher in years with higher than average rainfall; the highest annual snakebite prevalence in our survey was 2.4 times the average. Mohapatra *et al.* [13] estimated the annual death rate within Tamil Nadu to be 3,100. They suggest that Tamil Nadu has more snakebite deaths than other Indian states (4.7/100,000 compared with an average of 4.1/100,000). Their estimate is part of the Million Deaths Study within India as a whole, but within Tamil Nadu only a small sample of 38 deaths due to snakebite in 2001–2003 was used in that study. Interestingly, our data suggest that the number of bites is only 11 times the number of deaths, which is considerably lower than the ratio of 64 bites/death suggested by Mohapatra *et al*. [13] based only on hospital data. Hospital records, however, are often incomplete and not all victims attend hospital, either because they do not seek medical treatment, or because they die before hospital intervention is possible. Rahman *et al*. [21] also estimated around 100 non-fatal bites for each death in a survey of rural Bangladesh.

Conducting this type of household survey within selected regions would be necessary to confirm whether the observations of this study may be extrapolated to the whole state of Tamil Nadu. A considerable level of migration was evident from the study villages to urban areas within the last 10 years, as members of the population seek to gain access to better employment and education. The figures reached within this study may therefore represent an underestimation.

Consistent with other studies [4], [11], [21], our data indicate that immediate first aid often takes the form of traditional treatments. However this seems to occur less frequently than in other South Asian countries; for example 90% of victims in Nepal [11] and 98% of victims in Bangladesh [22] were treated with tourniquets, compared with just 20% in our survey. Nevertheless such treatments are contra-indicated, with the only recommended first aid being immobilization, though this requires equipment and training and is unsuitable for viper and cobra bites [4], [11], [23], [24]. In contrast to Bangladesh, where only 10% of victims seek hospital treatment, the majority of victims in our survey went to hospital, although over a quarter used further traditional treatments either instead or in addition. Consistent with other reports, the clinicians discouraged this and encouraged victims to seek medical treatment as soon as possible. The delays in arrival at hospital, possibly linked to patients first seeking locally available traditional treatments or to the distance from a health centre, caused complications; in Nepal the time taken to reach hospital was found to be a key determinant of mortality [25].

Given the extent of snakebite and the socio-economic effect on the lives of people in rural India, measures to reduce the incidence and to improve the treatment are clearly desirable. The socio-economic impacts that snakebites cause to victims are substantial. Treatment and living costs after the bite vary widely from person to person, and we have obtained as precise a range of costs as possible. Beginning from one-off direct cost (Rs1000–Rs350,000) to long term costs (up to Rs400,000) endanger the livelihood of the family. The type of venomous snake responsible for envenomation is also a factor in the extent of socio-economic effects to the victim and their family. For example, when Russell’s or saw scaled viper bites occurred, they caused severe bleeding disorders including cerebral haemorrhage (in one victim) and necrosis at the bite site, and these resulted in blood or plasma transfusion and/or skin grafts and major surgery. Similarly, three of the victims identified in this study were bitten by kraits, and these were misidentified as brain deaths due to the lack of fang marks or any other signs for a typical snakebite. Hence, inappropriate treatments were provided. Elapid bites frequently cause severe respiratory distress/failure resulting in a requirement for ventilator use and multi-speciality hospitals. These complications from specific snakes have further increased the treatment costs for the victims.

The clinicians that we interviewed in this study (data not shown) emphasized the need for reduction in the incidence of snakebite by raising community awareness of the risks, and prevention by wearing appropriate footwear. These measures have been suggested by other researchers [4], [11], and the WHO’s latest guidelines for the management of snakebite in South East Asia recognise that community education is the most effective preventive measure [26]. The clinicians also recommended improvements in the training of medical personnel in rural areas and in the education of medical students. This would enable correct administration of the locally available ASV requested by the public and rapid referral of patients to more distant hospitals where necessary. The victims also suggested educating the community to enable them to administer first aid and making the availability of first aid kits in the rural community centres for easy and immediate access. Standard protocols and tools for diagnosis and treatment of snakebite were recommended by the clinicians. This has been recommended by others [11], [12] and recently updated (2010) WHO guidelines have been published [26], however even in the private hospitals staffed by the clinicians interviewed in our study these were not available. Finally, improvements to the currently available antivenoms either in terms of reduced side effects or improved efficacy would be valuable. Antivenoms against specific snake venoms are not currently available in India, and the available polyvalent ASV may not be effective against bites from some snakes (e.g. hump-nosed pit viper and Levantine viper) more recently recognised to be of medical significance. Thus, production of antivenom against these snakes must be accelerated for immediate use. Research on the potential application of inhibitors (synthetic or from medicinal plant compounds) of venom enzymes may result in improved and more generic or cross-species effective therapy with reduced side effects in comparison to ASV.

This study emphasizes the extent of snakebite incidence and its socio-economic effects on the rural population of Tamil Nadu, India. We have investigated the issues associated with prevention and treatment by consulting the general population, the victims of snakebite and the clinicians involved in treatment. As researchers we are also keenly aware of the issues associated with understanding the components of snake venom in order to assist development of new treatments. We hope that this study will provide the incentive for researchers, the general public and clinicians to work together to achieve the key initiatives of the global snakebite initiative [15]: improved community education, improved education of medical personnel and improved research on efficacy and safety of antivenom.

## Supporting Information

Study Material S1 **Questionnaire used to interview the victims.** A detailed questionnaire was devised to ask victims about the circumstances of the snakebite, the method of treatments obtained and its socio-economic impacts to the family.
(DOC)
