# Supplementary material for: Snakebite and Its Socio-Economic Impact on the Rural Population of Tamil Nadu, India
Source: PLoS One. 2013 Nov 21;8(11):e80090. doi: 10.1371/journal.pone.0080090 (PMC3836953; doi:10.1371/journal.pone.0080090)
Supplement: Study Material S1 — Questionnaire used to interview the victims. A detailed questionnaire was devised to ask victims about the circumstances of the snakebite, the method of treatments obtained and its socio-economic impacts to the family. (DOC) [file pone.0080090.s001.doc]

**Victim’s questionnaire**

**Interviewee’s details**

Name:

Address:

Is the interviewee the victim of the snake bite or not?

If not, what is the relationship to the snake bite victim?

**Personal details of the snake bite victim**

First Name and First Letter of Last Name (if not the same as interviewee):

Gender: M/ F

Village:

Occupation:

Is victim still alive?

If not, did he/ she die because of the snake bite?

If not, what caused the death?

How many times has the victim been bitten by a snake?

**Snake bite-incident details (questions between * and * to be asked for each incident)**

Age of victim when incident occurred:*****

Where was the victim when the snake bite occurred?

What was the victim doing when the bite happened?

What time of day did the snake bite occur?

What time of year did the snake bite occur?

Which part of the body was bitten?

Was the snake identified and if so, what type of snake was it?

How big was the snake?

Did you kill the snake to take to the hospital?

What effects did the snake bite have on the victim?

**Treatments**

1. First aid

What was the first aid given?

How long from snake bite to first aid?

Who gave the first aid?

1. Traditional Treatments

Did you seek help from traditional healers?

How long after the snake bite?

What made you go there?

What treatment did they give you?

How long did you have treatment with them?

How much did the treatment cost?

Did this cure the effects of the snake bite?

1. Hospital Treatment

Did you go the hospital?

How long after the snake bite?

Where was the hospital?

Hospital name

Do you know the name of the doctor who treated you mainly?

Distance from the incident area to the hospital?

How long did you take to reach the hospital?

What did the doctor ask you regarding the incident?

Do you know what treatments they gave you?

How long did you stay in the hospital?

How much did the treatment cost?

**Economical issues**

How much money did you spend in total for the treatment?

Did you have enough money to pay the hospital?

Did you get any loan?

How much was the loan?

Did you get any support from government or society or any other charities?

How did this snake bite affect your daily life?

How long was it before you were able to work again?

How did you feel about working in the same land again?

Did the bite affect your working ability?

Did this incident affect your family? *****

**Victim’s expectations**

What do you think about the current treatments for snake bites?

Do you think the treatment has to be improved?

Do you think the treatment should be available closer to your village?

Would you expect any help from government? And what would that be?

**Prevention**

Do you know how to prevent snake bites hereafter?

What measures are you currently taking to protect yourself from snake bite?

What advice would you give to others to prevent snake bites?

Would you be happy to answer further questions in the future should the need arise?
